# The effect of anthocyanins supplementation on liver enzymes: A systematic review and meta‐analysis of randomized clinical trials

**DOI:** 10.1002/fsn3.2278

**Published:** 2021-05-06

**Authors:** Zohreh Sadat Sangsefidi, Hassan Mozaffari‐Khosravi, Sahar Sarkhosh‐Khorasani, Mahdieh Hosseinzadeh

**Affiliations:** ^1^ Nutrition and Food Security Center School of Public Health Shahid Sadoughi University of Medical Sciences Yazd Iran; ^2^ Department of Nutrition School of Public Health Shahid Sadoughi University of Medical Sciences Yazd Iran

**Keywords:** anthocyanins, liver enzymes, meta‐analysis, systematic review

## Abstract

This systematic review and meta‐analysis aimed to assess effect of consuming anthocyanins (ACNs; pure ACNs or products containing ACNs) on liver enzymes levels including alanine aminotransferase (ALT), aspartate aminotransferase (AST), and gamma‐glutamyl transferase (GGT). Although no significant impact was detected on the liver enzymes, a significant reduction was observed on ALT (WMD = −4.932 U/L, 95% CI = −9.848 to −0.015, *p* = .049) and AST (WMD = −3.464 U/L, 95% CI = −6.034 to −0.894, *p* = .008) in the studies that examined them as primary outcomes. A significant decrease was found on AST among the healthy subjects (WMD = −4.325 U/L, 95% CI = −8.516 to −0.134, *p* = .043) and in the studies that used products containing ACNs as intervention (WMD = −2.201 U/L, 95% CI = −4.275 to −0.127, *p* = .037). Although no significant relation was detected between ACNs dosage and the liver enzymes, significant associations were found between the duration of trial with ALT (ALT: slope: 0.09, 95% CI = 0.040 to 0.139, *p* = .0003) and AST (slope: 0.076, 95% CI = 0.037 to 0.115, *p* = .0001). In conclusion, although ACNs had no significant effect on the liver enzymes, a significant decrease was discovered on ALT and AST in the studies that evaluated them as primary outcomes. A significant reduction was observed in AST in the healthy individuals and in the studies used products containing ACNs as intervention. Significant relations were also found between the duration of trial with ALT and AST. Further studies are required to confirm these results.

## Graphical abstract

This systematic review and meta‐analysis indicated no significant impact on ALT, AST, and GGT levels. However, a significant reduction was found on ALT and AST in the studies which examined liver enzymes as primary outcomes. Moreover, a significant decrease was observed on AST among healthy subjects and in the studies with products rich in ACNs as intervention.

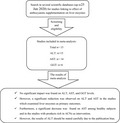

## 1 INTRODUCTION

Anthocyanins (ACNs) are a group of water‐soluble natural pigments. They belong to flavonoids and are found in different plant sources such as fruits, vegetables, grains, and cereals in red, purple, and blue (Bueno et al., 2012; Sangsefidi et al., 2018). Evidences indicated beneficial impacts of ACNs on disorders involving inflammatory and oxidative processes such as cardiovascular diseases (Wallace et al., 2016), type 2 diabetes (Sancho & Pastore, 2012), metabolic syndrome (Tsuda, 2008), and dyslipidemia (Liu et al., 2016). In addition, useful effects of these components were reported on glycemic control (Daneshzad et al., 2019; Yang, Ling, Du, et al., 2017) and lipid profile (Daneshzad et al., 2019; Shah & Shah, 2018; Yang, Ling, Du, et al., 2017).

Based on the literature, increase of the serum liver enzymes can be related to the injury of liver cells due to some factors including oxidative stress, and inflammation or increased fat storage in the liver following insulin resistance (Ahn et al., 2014; Bonnet et al., 2011; Suda et al., 2008; Zhang et al., 2010). The results of some studies indicated that ACNs had beneficial impacts on liver disorders such as NAFLD, and borderline hepatitis, as well as liver enzymes (Chang et al., 2014; Oki et al., 2016; Suda et al., 2008; Zhang et al., 2015). The protective effects of ACNs on liver were attributed to anti‐inflammatory and antioxidant roles of these compounds, improvement of insulin resistance, lipid profile, and glycemic control (Chang et al., 2014; Guo et al., 2014; Oki et al., 2016; Suda et al., 2008; Zhang et al., 2015). However, findings of surveys over the effect of ACNs on liver enzymes levels are inconsistent and controversial. For example, in a research on NAFLD patients, supplementation with purified ACNs for 12 weeks decreased the alanine aminotransferase (ALT) levels (Zhang et al., 2015). In addition, consuming the purple sweet potato extract beverage for 8 weeks was related to decreased liver enzymes levels among healthy Caucasians with borderline hepatitis (Oki et al., 2016). Similarly, a significant reduction was found in concentration of liver enzymes following the consumption of a sweet potato extract beverage for 12 weeks in healthy men with borderline hepatitis (Suda et al., 2008). Consumption of freeze‐dried blueberries for 8 weeks was associated with reduced concentrations of ALT and aspartate aminotransferase (AST) in men with type 2 diabetes (Stote et al., 2020). However, some other studies showed that ACNs had no significant effect on liver enzyme; for instance, intake of an elderberry extract in postmenopausal healthy women (Curtis et al., 2009) and pure ACNs in patients with pre‐diabetes (Yang, Ling, Yang, et al., 2017) did not have any significant effect on liver enzymes. Moreover, ingestion of Hibiscus sabdariffa extract did not affect liver enzymes among adults with NAFLD significantly (Chang et al., 2014). To the best of our knowledge, no systematic review and meta‐analysis have ever been carried out on this issue. Therefore, the present systematic review and meta‐analysis were conducted to prepare a more accurate estimate of the overall effect of ACNs on liver enzymes. Our objective was to investigate the impact of supplementation with ACNs (pure ACNs or products rich in ACNs) on liver enzymes levels including alanine aminotransferase (ALT), aspartate aminotransferase (AST), and gamma‐glutamyl transferase (GGT).

## 2 MATERIAL AND METHODS

### 2.1 Search strategy

The current systematic review and meta‐analysis were carried out according to the Preferred Reporting Items for Systematic Reviews and Meta‐Analyses Guidelines (PRISMA) (Moher et al., 2015). This study was registered at crd.york.ac.uk/Prospero as CRD42020150700. Several databases including PubMed, ISI Web of Science, Scopus, and Google Scholar were searched up to 25 Jun 2020 without any restrictions to identify eligible researches. Medical Subject Heading (MeSH) terms and non‐MeSH terms were applied to evaluate the impact of ACNs on liver enzymes levels. The following keywords were used to search: ((anthocyanins OR "anthocyanin extract" OR cyanidin OR pelargonidin OR pelargonidin OR delphinidin OR peonidin OR petunidin) AND (liver OR "liver enzyme" OR *transaminase* OR *aminotransferase* OR *transpeptidase* OR "alanine transaminase OR "alanine aminotransferase" OR ALT OR SGPT OR "aspartate aminotransferases" OR transaminases OR AST OR SGOT OR "alkaline phosphatase" OR ALP OR "gamma‐glutamyltransferase" OR "gamma glutamyltransferase" OR GGT OR "gammaglutamyltransferase" OR "lactate dehydrogenase" OR "L‐lactate dehydrogenase" OR "dehydrogenase L‐lactate" OR "dehydrogenase lactate" OR LDH OR "AST‐to‐ALT ratio" OR "ALT to AST ratio" OR "liver enzyme abnormality" OR "liver enzyme activity" OR "liver function tests" OR LEA OR "AST/ALT")).

Furthermore, to ensure about the comprehensiveness of searches, references of the included studies were checked for further possible sources.

### 2.2 Selection criteria

The selected surveys had the following criteria: (a) had RCT design; (b) assessed the effect of pure ACNs or products rich in ACNs including extracts, beverages, powders or juices on liver enzymes levels versus placebo/control; (c) presented the administered ACNs dosage or reported a quantifiable ACNs content for products rich in ACNs; (d) included participants aged ≥18 years; and (e) reported sufficient data for liver enzymes levels. We excluded studies if they had additional intervention other than pure ACNs or products rich in ACNs such as additional supplement or additional herbal products.

### 2.3 Study selection

**FIGURE 1:**
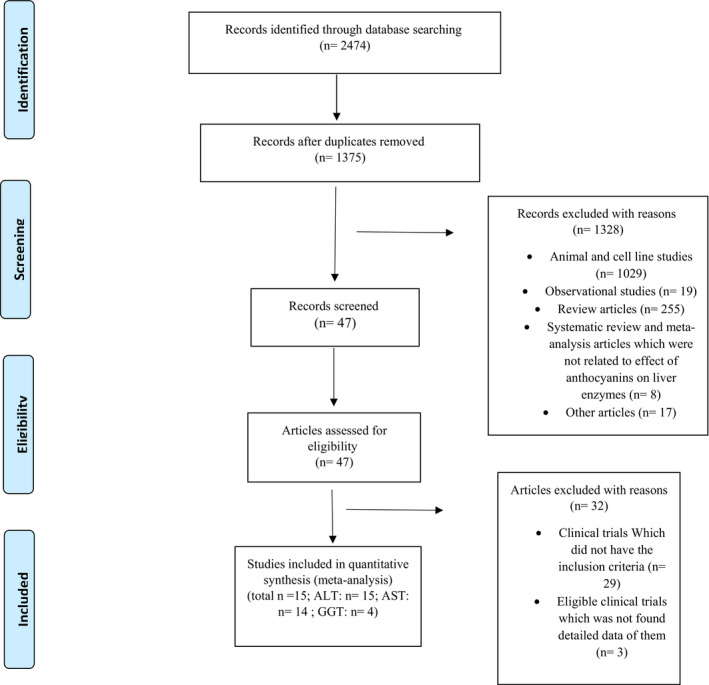
Flow chart of studies selection process

The initial screening was conducted by two independent researchers (ZS.S and S.K‐H), who studied the articles' titles and abstracts. Then, the full texts of all related trials were evaluated via reviewers to select the articles about the effect of ACNs (pure ACNs or products rich in ACNs including extracts, beverages, powders or juices) on liver enzymes. Eventually, any possible disagreement was negotiated and solved by consultation with other researchers (M.H and H.M‐K; Figure 1).

### 2.4 Data extraction

Data were extracted from the selected studies by the following criteria: authors' family names; publication year; sample size; loss to follow‐up; intervention type and its dosage; study duration; cross‐over or parallel study design; participants' gender, age, and health status; mean and standard deviation (*SD*) of liver enzymes concentration (serum or plasma) at the beginning and at the end of the trial, as well as the mean changes and *SD*s of biomarkers' levels.

### 2.5 Risk of bias assessment

Risk of bias assessment of the included researches was assessed based on the Cochrane criteria (Higgins & Green, 2011). The following items were considered for evaluation of risk of bias of each study: (a) random sequence generation; (b) allocation concealment; (c) blinding of participants and personnel; (d) blinding of outcome assessment; (e) incomplete outcome data; (f) selective outcome reporting; and (g) other potential sources of bias. Based on the Cochrane Handbook recommendations, trials were rated on each the item as “yes” demonstrating low risk of bias, “no” indicating high risk of bias or “unclear” when the risk of bias was unclear or unknown.

### 2.6 Data synthesis and analysis

Difference in means was defined as effect sizes. Weighted mean differences (WMDs) were calculated as follows: mean divided by the standard deviation of a difference between two random values each from one of two groups (Higgins, 2011). In trials that the standard error (*SE*) value was reported, *SD* was obtained using the following formula: *SD *= *SE* × √*n* (*n *= number of participants in each group). The random‐effects model was applied to compute the WMDs with 95% confidence intervals (CIs) for performing the meta‐analysis, which took the between‐study heterogeneity into account (Borenstein et al., 2009). Heterogeneity of trials was also assessed using Cochran's *Q* test and *I*‐squared (*I* ^2^) statistic. Heterogeneity was defined as follow: *Q* statistic *p* value of <.1; weak heterogeneity: *I* ^2^ = 25–50, relatively high heterogeneity: *I* ^2^ = 50–75, high heterogeneity: *I* ^2^ = 75–100 (Higgins & Thompson, 2002; Sangsefidi et al., 2020). Subgroup analysis was also accomplished to identify the possible sources of heterogeneity among the selected studies. Since type of intervention (pure ACNs or products rich in ACNs including extracts, beverages, powders, or juices), the administered ACNs dosage, health status of participants (healthy, unhealthy [liver disease, other disease]), trial duration, and assessing liver enzymes as primary or secondary outcomes might have influenced on the results regarding to impact of ACNs, subgroup analysis was carried out based on these variables. Moreover, publication bias was assessed by evaluation of the funnel plot and asymmetry tests including Begg's rank correlation test and Egger's regression test (using *p* value of <.05) (Duval & Tweedie, 2000). Sensitivity analysis was also conducted to specify the impact of a specific study or a particular group of studies via individual removal of each trial or a specific group and recalculation of the pooled estimates.

Moreover, meta‐regression was conducted to evaluate relation of the estimated effect size with ACNs dosage and trial duration.

Statistical analyses were conducted using STATA software, version 11.2 (STATA Corp.). Statistically significant levels were considered as *p* < .05.

## 3 RESULTS

### 3.1 Study selection and characteristics

Our electronic search of several databases including PubMed, Web of Science, Scopus, and Google scholar resulted in 2,474 articles. 1,375 studies remained after excluding duplicates. Of this numbers, 1,358 researches were excluded since they were not clinical trials (*n* = 1,328) or did not meet the inclusion criteria (*n* = 32). Eventually, 15 surveys met the inclusion criteria and entered in meta‐analysis (Figure 1). We investigated 18 studies in our systematic review as we could not find full text of one article (Kano et al., 2018). For meta‐analysis, we entered only 15 studies since we did not achieve detailed data of two researches (Hassellund et al., 2013; Kianbakht & Hashem‐Dabaghian, 2019) and exclude one study (Guo et al., 2014) because it affected the results due to cross‐over design. Characteristics of the included trials are presented in Tables 1, 2, 3. All researches were published from 2008 to 2020. The total number of participants in the included trials who completed the surveys was 1,028 (*n* = 514 in the intervention group, *n* = 514 in the placebo group). Design of all RCTs was parallel except two studies (Guo et al., 2014; Hassellund et al., 2013) that had cross‐over design. Furthermore, most of participants were patients with different diseases such as NAFLD (Chang et al., 2014; Guo et al., 2014; Zhang et al., 2015), pre‐diabetes, or diabetes type 2 (Kianbakht et al., 2013; Soltani et al., 2015; Stote et al., 2020; Yang, Ling, Yang, et al., 2017), dyslipidemia (Kianbakht et al., 2014; Qin et al., 2009; Soltani et al., 2014), overweight and obese adults (Wright, Netzel, & Sakzewski, 2013), and pre‐hypertension (Hassellund et al., 2013) or hypertension (Kianbakht & Hashem‐Dabaghian, 2019; Mohtashami et al., 2019). Nevertheless, three surveys (Curtis et al., 2009; Oki et al., 2016; Suda et al., 2008) had been studied healthy subjects. In addition, only five studies (Chang et al., 2014; Oki et al., 2016; Stote et al., 2020; Suda et al., 2008; Zhang et al., 2015) evaluated liver enzymes as primary outcomes among all researches. Trials durations varied from 28 to 90 days. Dose of administered ACNs was also from 0.77 to 640 mg/day.

**TABLE 1:** The characteristics of the included studies regarding to the effect of anthocyanins on Alanine Aminotransferase (ALT) levels

Study	Study design	Study population	Type of intervention	Anthocyanins dose (mg/day)	Trial duration	ALT levels (U/L)	
						Baseline	End of trial
1. Oki et al. (2016)	Randomized double‐blind, placebo‐controlled clinical trial (parallel)	Healthy Caucasians with borderline hepatitis. Total *n* = 40. But *n* = 37 (18 in PSP group and 19 in placebo group) completed the study	Anthocyanin‐rich purple‐ sweet potato (PSP) extract beverage or placebo	531	8 weeks	PSP group: 23.3 ± 15.7 Placebo group: 19.2 ± 9.0	PSP group: Not reported Placebo group: Not reported
2. Kianbakht et al. (2013)	Randomized double‐blind, placebo‐controlled clinical trial (parallel)	Patients with diabetes type 2 Total *n* = 86. But *n* = 74 (*n* = 37 in each group) completed the study.	Whortleberry fruit extract or placebo	9.088	8 weeks	Whortleberry group: 21.5 ± 8.3 Placebo group: 19.3 ± 6.7	Whortleberry group: 26.7 ± 9 Placebo group: 25.8 ± 11.4
3. Suda et al. (2008)	Randomized double‐blind Placebo‐controlled clinical trial (parallel)	Healthy men with borderline hepatitis. Total *n* = 48. But *n* = 38 (*n* = 20 in PSP group and *n* = 18 in placebo group) completed the study	Purple sweet potato (PSP) extract beverage or placebo	400.6	12 weeks	PSP group: **51.3 (5) Placebo group: **46.5 (5.1)	PSP group: Not reported Placebo group: Not reported
4. Qin et al. (2009)	Randomized double‐blind Placebo‐controlled clinical trial (parallel)	Patients with dyslipidemia. Total *n* = 120. *n* = 60 in each group completed the study	Purified anthocyanins or placebo	320	12 weeks	Anthocyanins group: 17.5 ± 7.4 Placebo group: 19.4 ± 8.1	Anthocyanins group: 17.1 ± 6.6 Placebo group: 18.8 ± 8.1
5. Curtis et al. (2009)	Randomized double‐blind Placebo‐controlled clinical trial (parallel)	Healthy postmenopausal women. Total *n* = 56. *N* = 26 completed the study	Elderberry extract or placebo	500	12 weeks	Elderberry group: 21.3 ± 10.8 Placebo group: 18.9 ± 6.7	Elderberry group: 18.7 ± 5.8 Placebo group: 17.6 ± 7.5
6. Chang et al. (2014)	Randomized double‐blind Placebo‐controlled clinical trial (parallel)	Subjects with a BMI higher than 27 and NAFLD. Total *n* = 40. But *n* = 36 (*n* = 19 in HSE group and *n* = 17 in placebo group) completed the study	Hibiscus sabdariffa extracts (HSE) or placebo	67.5	12 weeks	HSE group: 57.21 ± 35.45 Placebo group: 35.47 ± 20.04	HSE group: 55.63 ± 35.62 Placebo group: 28.94 ± 11.69
7. Kianbakht et al. (2014)	Randomized double‐blind Placebo‐controlled clinical trial (parallel)	Patients with hyperlipidemia. Total *n* = 105. But *n* = 80 (*n* = 40 in each group) completed the study	Whortleberry fruit extract or placebo	7.35	2 months	Whortleberry group: 24.42 ± 10.05 Placebo group: 27.07 ± 13.63	Whortleberry group: 23.37 ± 10.42 Placebo group: 22.1 ± 10.65
8. Zhang et al. (2015)	Randomized double‐blind Placebo‐controlled clinical trial (parallel)	Patients with NAFLD. Total *n* = 74. *n* = 37 in each group completed the study	Purified anthocyanins or placebo	320	12 weeks	Anthocyanins group: 36 ± 20.6 Placebo group: 34 ± 20	Anthocyanins group: 28 ± 9.6 Placebo group: 33 ± 17.03
9. Yang, Ling, Yang, et al. (2017)	Randomized double‐blind Placebo‐controlled clinical trial (parallel)	Patients with pre‐diabetes or early untreated diabetes. Total *n* = 160. *n* = 80 in each group completed the study	Purified anthocyanins or placebo	320	12 weeks	Anthocyanins group: 21.58 ± 8.66 Placebo group: 23.63 ± 8.78	Anthocyanins group: 20.40 ± 9.30 Placebo group: 21.01 ± 9.2
10. Soltani et al. (2014)	Randomized double‐blind Placebo‐controlled clinical trial (parallel)	Hyperlipidemic patients. Total *n* = 54. But *n* = 50 (25 in each group) completed the study	*Vaccinium arctostaphylos* fruit extract or placebo	90	4 weeks	*Vaccinium* arctostaphylos group: 22.48 ± 10.88 Placebo group: 24.72 ± 9.12	*Vaccinium* arctostaphylos group: 20.88 ± 11.51 Placebo group: 24.24 ± 8.18
11. Soltani et al. (2015)	Randomized double‐blind Placebo‐controlled clinical trial (parallel)	Diabetic Patients. Total *n* = 60. *n* = 30 in each group completed the study	Cornus mas L. Fruit Extract or placebo	600	6 weeks	Cornus mas L. group: 16.43 ± 9.61 Placebo group: 17.46 ± 8.16	Cornus mas L. group: 16.70 ± 6.47 Placebo group: 17.86 ± 7.44
12. Kianbakht and Hashem‐Dabaghian (2019)	Randomized double‐blind Placebo‐controlled clinical trial (parallel)	Obese hypertensive outpatients Total *n* = 112. But *n* = 100 (*n* = 50 in each group) completed the study	*Vaccinium arctostaphylos* extract or placebo	2.59	3 months	*Vaccinium arctostaphylos* group: Not reported Placebo group: Not reported	*Vaccinium arctostaphylos* group: Not reported Placebo group: Not reported
13. Hassellund et al. (2013)	Randomized double‐blind placebo‐controlled clinical trial (cross‐over)	Pre‐hypertensive men. Total *n* = 31. But *n* = 27 completed the study	Purified anthocyanin or placebo	640	12 weeks	Anthocyanins group: Not reported Placebo group: Not reported	Anthocyanin group: Not reported Placebo group: Not reported
14. Mohtashami et al. (2019)	Randomized double‐blind placebo‐controlled clinical trial (parallel)	Hypertensive hyperlipidemic type 2 diabetic patients. Total *n* = 103 (*n* = 51 in extract and *n* = 52 in placebo group). But *n* = 100 (*n* = 50 in each group) completed the study	*Vaccinium arctostaphylos* leaf extract or placebo	0.77	2 months	*Vaccinium arctostaphylos* group: 15.9 ± 8.9 Placebo group: 17.2 ± 6.9	*Vaccinium arctostaphylos* group: 14.8 ± 10.2 Placebo group: 16.5 ± 9.0
15. Asgary et al. (2016)	Randomized double‐blind placebo‐controlled clinical trial (parallel)	Hyperlipidemic patients. Total *n* = 46 (23 in each group). But *n* = 40 (*n* = 20 in each group) completed the study	*Vaccinium arctostaphylos* fruit extract or placebo	1.6	4 weeks	*Vaccinium arctostaphylos* group: 22.48 ± 10.88 Placebo group: 24.72 ± 9.12	*Vaccinium arctostaphylos* group: 20.88 ± 11.51 Placebo group: 24.24 ± 8.18
16. Wright et al. (2013)	Randomized double‐blind placebo‐controlled clinical trial (parallel)	Healthy male. Total *n* = 16. *n* = 16 (*n* = 8 in each group) completed the study	Dried purple carrot powder or placebo	118.5	4 weeks	Dried purple carrot group: 25.6 ± 6.5 Placebo group: 32.9 ± 15.4	Dried purple carrot group: 35.8 ± 15.5 Placebo group: 31.3 ± 19.9
17. Guo et al. (2014)	Randomized double‐blind, placebo‐controlled clinical trial (cross‐over)	Young adults with NAFLD. Total *n* = 44. But *n* = 44 in barberry group and *n* = 43 in placebo group completed the study	Bayberry juice or placebo	417.5	10 weeks	Bayberry group: 25.1 ± 20.37 Placebo group: 23.8 ± 14.29	Bayberry group: 24.1 ± 22.59 Placebo group: 22.2 ± 14.81
18. Stote et al. (2020)	Randomized double‐blind placebo‐controlled clinical trial (parallel)	Men with diabetes type 2. *n* = 55 (*n* = 27 in blue berry group and *n* = 28 in placebo group). But *n* = 26 in each group completed the study	Freeze‐dried blueberry or placebo	261.8	8 weeks	Blueberry group: 36.5 ± 2.7 Placebo group: 39.9 ± 3.8	Blueberry group: 35.6 ± 1.5 Placebo group: 48.3 ± 2.9

Note

All values expressed as mean ± standard deviation (*SD*) except **for study number 3: Values presented as mean (95% CI).

**TABLE 2:** The characteristics of the included studies regarding to the effect of anthocyanins on Aspartate Aminotransferase (AST) levels

Study	Study design	Study population	Type of intervention	Anthocyanins dose (mg/day)	Trial duration	AST levels (U/L)	
						Baseline	End of trial
1. Oki et al. (2016)	Randomized double‐blind, placebo‐controlled clinical trial (parallel)	Healthy Caucasians with borderline hepatitis. Total *n* = 40. But *n* = 37 (18 in PSP group and 19 in placebo group) completed the study	Anthocyanin‐rich purple‐ sweet potato extract (PSP) beverage or placebo	531	8 weeks	PSP group: 31.1 ± 14.1 Placebo group: 25.6 ± 5.0	PSP group: Not reported Placebo group: Not reported
2. Kianbakht et al. (2013)	Randomized double‐blind, placebo‐controlled clinical trial (parallel)	Patients with diabetes type 2 Total *n* = 86. But *n* = 74 (*n* = 37 in each group) completed the study	Whortleberry fruit extract or placebo	9.088	8 weeks	Whortleberry group: 22 ± 6.6 Placebo group: 20.7 ± 5.7	Whortleberry group: 25.3 ± 7 Placebo group: 22.8 ± 6.2
3. Suda et al. (2008)	Randomized double‐blind placebo‐controlled clinical trial (parallel)	Healthy men with borderline hepatitis. Total *n* = 48. But *n* = 38 (*n* = 20 in PSP group and *n* = 18 in placebo group) completed the study	Purple sweet potato (PSP) extract beverage or placebo	400.6	12 weeks	PSP group: **35.5 (2.2) Placebo group: **32.5 (2.3)	PSP group: Not reported Placebo group: Not reported
4. Qin et al. (2009)	Randomized double‐blind placebo‐controlled clinical trial (parallel)	Patients with dyslipidemia. Total *n* = 120. *n* = 60 in each group completed the study	Purified anthocyanins or placebo	320	12 weeks	Anthocyanins group: 20.3 ± 6.4 Placebo group: 20.4 ± 6.6	Anthocyanins group: 18.7 ± 3.8 Placebo group: 19.8 ± 6.6
5. Chang et al. (2014)	Randomized double‐blind placebo‐controlled clinical trial (parallel)	Subjects with a BMI higher than 27 and NAFLD. Total *n* = 40. But *n* = 36 (*n* = 19 in HSE group and *n* = 17 in placebo group) completed the study	Hibiscus sabdariffa extracts (HSE) or placebo	67.5	12 weeks	HSE group: 33.05 ± 17.82 Placebo group: 23.18 ± 9.34	HSE group: 31.11 ± 17.25 Placebo group: 19.53 ± 3.97
6. Kianbakht et al. (2014)	Randomized double‐blind placebo‐controlled clinical trial (parallel)	Patients with hyperlipidemia. Total *n* = 105. But *n* = 80 (*n* = 40 in each group) completed the study	Whortleberry fruit extract or placebo	7.35	2 months	Whortleberry group: 21.75 ± 6.40 Placebo group: 23.82 ± 7.51	Whortleberry group: 24.40 ± 7.56 Placebo group: 27.95 ± 13.68
7. Zhang et al. (2015)	Randomized double‐blind placebo‐controlled clinical trial (parallel)	Patients with NAFLD. Total *n* = 74. *n* = 37 in each group completed the study	Purified anthocyanins or placebo	320	12 weeks	Anthocyanins group: 28 ± 6.6 Placebo group: 26 ± 5.1	Anthocyanins group: 26.5 ± 9.6 Placebo group: 26 ± 9.6
8. Yang, Ling, Du, et al. (2017) and Yang, Ling, Yang, et al. (2017)	Randomized double‐blind placebo‐controlled clinical trial (parallel)	Patients with pre‐diabetes or early untreated diabetes. Total *n* = 160. *n* = 80 in each group completed the study	Purified anthocyanins or placebo	320	12 weeks	Anthocyanins group: 22.05 ± 5.68 Placebo group: 22.78 ± 5.36	Anthocyanins group: 23.20 ± 6.15 Placebo group: 22.71 ± 5.66
9. Soltani et al. (2014)	Randomized double‐blind placebo‐controlled clinical trial (parallel)	Hyperlipidemic patients. Total *n* = 54. But *n* = 50 (25 in each group) completed the study	*Vaccinium* *arctostaphylos* fruit extract or placebo	90	4 weeks	*Vaccinium arctostaphylos* group: 21.60 ± 7.77 Placebo group: 23.20 ± 7.48	*Vaccinium arctostaphylos* group: 20.60 ± 7.94 Placebo group: 23.68 ± 8.48
10. Soltani et al. (2015)	Randomized double‐blind placebo‐controlled clinical trial (parallel)	Diabetic Patients. Total *n* = 60. *n* = 30 in each group completed the study	Cornus mas L. Fruit Extract or placebo	600	6 weeks	Cornus mas L. group: 22.76 ± 6.75 Placebo group: 22.16 ± 9.83	Cornus mas L. group: 23.36 ± 7.07 Placebo group: 23.76 ± 14.13
11. Kianbakht and Hashem‐Dabaghian (2019)	Randomized double‐blind placebo‐controlled clinical trial (parallel)	Obese hypertensive outpatients Total *n* = 112. But *n* = 100 (*n* = 50 in each group) completed the study	*Vaccinium arctostaphylos* extract or placebo	2.595 ± 0.009	3 months	*Vaccinium arctostaphylos* group: Not reported Placebo group: Not reported	*Vaccinium arctostaphylos* group: Not reported Placebo group: Not reported
12. Hassellund et al. (2013)	Randomized double‐blind placebo‐controlled clinical trial (cross‐over)	Pre‐hypertensive men. Total *n* = 31. But *n* = 27 completed the study	Purified anthocyanin or placebo	640	12 weeks	Anthocyanins group: Not reported Placebo group: Not reported	Anthocyanin group: Not reported Placebo group: Not reported
13. Mohtashami et al. (2019)	Randomized double‐blind placebo‐controlled clinical trial (parallel)	Hypertensive hyperlipidemic type 2 diabetic patients. Total *n* = 103 (*n* = 51 in extract and *n* = 52 in placebo group). But *n* = 100 (*n* = 50 in each group) completed the study	*Vaccinium arctostaphylos* leaf extract or placebo	0.77	2 months	*Vaccinium arctostaphylos* group: 25.2 ± 6.3 Placebo group: 21.2 ± 7.2	*Vaccinium arctostaphylos* group: 23.2 ± 12.6 Placebo group: 24.3 ± 12.2
14. Asgary et al. (2016)	Randomized double‐blind placebo‐controlled clinical trial (parallel)	Hyperlipidemic patients. Total *n* = 46 (23 in each group). But *n* = 40 (*n* = 20 in each group) completed the study	*Vaccinium arctostaphylos* fruit extract or placebo	1.6	4 weeks	*Vaccinium arctostaphylos* group: 21.6 ± 7.7 Placebo group: 23.2 ± 7.48	*Vaccinium arctostaphylos* group: 20.6 ± 7.94 Placebo group: 23.68 ± 8.48
15. Wright et al. (2013)	Randomized double‐blind placebo‐controlled clinical trial (parallel)	Healthy male. Total *n* = 16. *n* = 16 (*n* = 8 in each group) completed the study	Dried purple carrot powder or placebo	118.5	4 weeks	Dried purple carrot group: 24.9 ± 6.1 Placebo group: 26.0 ± 9.0	Dried purple carrot group: 27.1 ± 10.7 Placebo group: 24.1 ± 11.6
16.Guo et al. (2014)	Randomized double‐blind, placebo‐controlled clinical trial (Cross‐over)	Young adults with NAFLD. Total *n* = 44. But *n* = 44 in barberry group and *n* = 43 in placebo group completed the study	Bayberry juice or placebo	417.5	10 weeks	Bayberry group: 19.8 ± 3.6 Placebo group: 21.0 ± 5.2	Bayberry group: 21.3 ± 4.1 Placebo group: 20.5 ± 4.1
17. Stote et al. (2020)	Randomized double‐blind placebo‐controlled clinical trial (parallel)	Men with diabetes type 2. *n* = 55 (*n* = 27 in blue berry group and *n* = 28 in placebo group). But *n* = 26 in each group completed the study	Freeze‐dried blueberry or placebo	261.8	8 weeks	Blueberry group: 23.5 ± 1.9 Placebo group: 24.9 ± 2.9	Blueberry group: 23.2 ± 1.4 Placebo group: 30.5 ± 2.7

Note

All values expressed as mean ± standard deviation (*SD*) except **for study number 3: Values presented as mean (95% CI).

**TABLE 3:** The characteristics of the included studies regarding to the effect of anthocyanins on Gamma‐glutamyl transferase (GGT) levels

Study	Study design	Study population	Type of intervention	Anthocyanins dose (mg/day)	Trial duration	GGT levels (U/L)	
						Baseline	End of trial
1. Oki et al. (2016)	Randomized double‐blind, placebo‐controlled clinical trial (parallel)	Healthy Caucasians with borderline hepatitis. Total *n* = 40. But *n* = 37 (18 in PSP group and 19 in placebo group) completed the study	Anthocyanin‐rich purple‐fleshed sweet potato (PSP) beverage or placebo	531	8 weeks	PSP group: 74.6 ± 35.1 Placebo group: 57.1 ± 16.7	PSP group: Not reported Placebo group: Not reported
2. Suda et al. (2008)	Randomized double‐blind placebo‐controlled clinical trial (parallel)	Healthy men with borderline hepatitis. Total *n* = 48. But *n* = 38 (*n* = 20 in PSP group and *n* = 18 in placebo group) completed the study	Purple sweet potato (PSP) beverage or placebo	400.6	12 weeks	PSP group: **103.6 (17.3) Placebo group: **91.6 (11.1)	PSP group: Not reported Placebo group: Not reported
3. Curtis et al. (2009)	Randomized double‐blind placebo‐controlled clinical trial (parallel)	Healthy postmenopausal women. Total *n* = 56. *N* = 26 completed the study	Elderberry extract or placebo	500	12 weeks	Elderberry group: 21.0 ± 14.2 Placebo group: 22.0 ± 16.3	Elderberry group: 18.3 ± 9.5 Placebo group: 19.0 ± 11.5
4. Chang et al. (2014)	Randomized double‐blind placebo‐controlled clinical trial (parallel)	Subjects with a BMI higher than 27 and NAFLD. Total *n* = 40. But *n* = 36 (*n* = 19 in HSE group and *n* = 17 in placebo group) completed the study	Hibiscus sabdariffa extracts (HSE) or placebo	67.5	12 weeks	HSE group: 49.26 ± 45.39 Placebo group: 40.88 ± 32.57	HSE group: 50.05 ± 40 Placebo group: 35.65 ± 26.08

Note

All values expressed as mean ± standard deviation (*SD*) except **for study number 2: Values presented as mean (95% CI).

### 3.2 Risk of bias of the included studies

Risk of bias assessment of the included studies has been shown in Table 4. Of all the trials (*n* = 18), only 11 studies (Asgary et al., 2016; Curtis et al., 2009; Hassellund et al., 2013; Kianbakht et al., 2013; Kianbakht et al., 2014; Kianbakht & Hashem‐Dabaghian, 2019; Mohtashami et al., 2019; Soltani et al., 2014; Stote et al., 2020; Yang, Ling, Yang, et al., 2017; Zhang et al., 2015) and seven researches (Hassellund et al., 2013; Kianbakht et al., 2013, 2014; Kianbakht & Hashem‐Dabaghian, 2019; Mohtashami et al., 2019; Yang, Ling, Yang, et al., 2017; Zhang et al., 2015) reported that had random sequence generation and allocation concealment respectively. Furthermore, all trials had blinding of participants and personnel. Risk of detection bias (blinding of outcome assessment) was also unclear among all surveys except in four trials (Hassellund et al., 2013; Kianbakht et al., 2013, 2014; Kianbakht & Hashem‐Dabaghian, 2019) which was low. In addition, risk of attrition bias (incomplete outcome data), reporting bias (selective reporting), and other potential sources of bias were low among all trials.

**TABLE 4:** Risk of bias assessment according to Cochrane criteria for the studies regarding to effect of anthocyanins on liver enzymes

Study (year)	Random Sequence Generation	Allocation concealment	Blinding of participants and personnel	Blinding of outcome assessment	Incomplete outcome data	Selective outcome reporting	Other sources of bias
Asgary et al. (2016)	L	U	L	U	L	L	L
Chang et al. (2014)	U	U	L	U	L	L	L
Curtis et al. (2009)	L	U	L	U	L	L	L
Guo et al. (2014)	U	U	L	U	L	L	L
Wright et al. (2013)	U	U	L	U	L	L	L
Hassellund et al. (2013)	L	L	L	L	L	L	L
Kianbakht et al. (2013)	L	L	L	L	L	L	L
Kianbakht et al. (2014)	L	L	L	L	L	L	L
Kianbakht and Hashem‐Dabaghian (2019)	L	L	L	L	L	L	L
Mohtashami et al. (2019)	L	L	L	U	L	L	L
Oki et al. (2016)	U	U	L	U	L	L	L
Qin et al. (2009)	U	U	L	U	L	L	L
Soltani et al. (2015)	U	U	L	U	L	L	L
Soltani et al. (2014)	L	U	L	U	L	L	L
Suda et al. (2008)	U	U	L	U	L	L	L
Yang, Ling, Du, et al. (2017) and Yang, Ling, Yang, et al. (2017)	L	L	L	U	L	L	L
Zhang et al. (2015)	L	L	L	U	L	L	L
Stote et al. (2020)	L	U	L	U	L	L	L

Abbreviations: H, high risk; L, low risk, U, unclear risk.

### 3.3 Effect of ACNs on ALT

**FIGURE 2:**
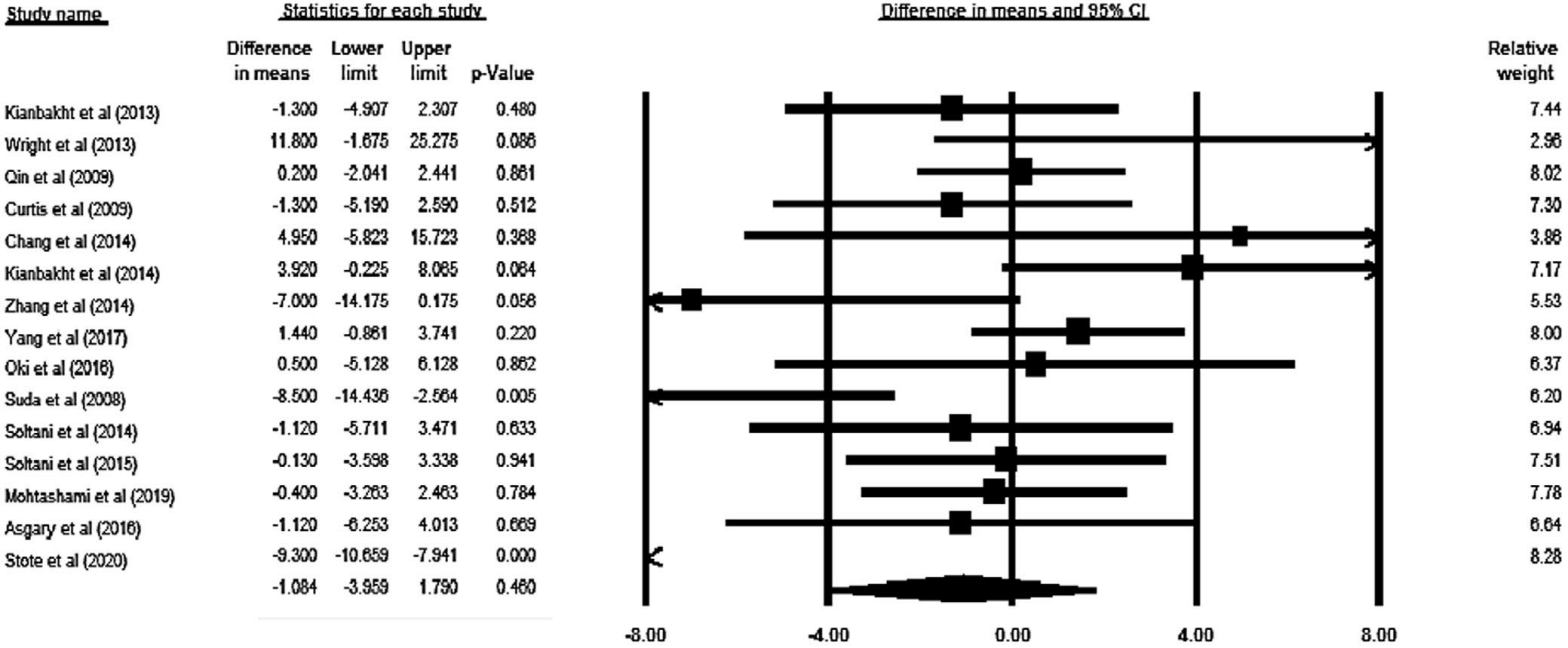
Forest plot illustrates weighted mean difference (represented by the black square) and 95% confidence interval (CI; represented by horizontal line) for Alanine Aminotransferase (ALT) concentration and anthocyanins. Weights are from random‐effects analysis. The area of the black square is proportional to the specific study weight to the overall meta‐analysis. The center of the diamond displays the pool weighted mean differences and its width shows the pooled 95% CI

Meta‐analysis of 15 eligible studies (total *n* = 1,028, intervention: *n* = 514, placebo: *n* = 514) demonstrated no significant impact on ALT concentrations after consuming ACNs (WMD = −1.084 U/L, 95% CI = −3.959 to 1.790, *p* = .460; Figure 2). This result was unchanged after removal of each study in the sensitivity analysis (Figure S1). The included trials had a significant heterogeneity (*p *˂ .0001, *I* ^2^ = 89.69). Based on subgroup analysis, the impact of ACNs did not significantly differ in the various doses (Dose ˃160 mg/day: WMD = −3.358 U/L, 95% CI = −7.986 to 1.269, *p* = .155; Dose ≤160 mg/day: WMD = 0.287 U/L, 95% CI = −1.234 to 1.808, *p* = .712; Table 5) and durations (Duration >56 days: WMD = −0.455 U/L, 95% CI = −2.629 to 1.719, *p* = .682; Duration ≤56 days: WMD = −1.279 U/L, 95% CI = −5.884 to 3.327, *p* = .586; Table 5). Moreover, ACNs had no significant effect among both healthy (WMD = −2.869 U/L, 95% CI = −7.701 to 1.963, *p* = .245; *I* ^2^ = 62.834; *Q* statistics (*p*) = .068) and unhealthy (liver disease: WMD = −1.728 U/L, 95% CI = −13.358 to 9.901, *p* = .771; *I* ^2^ = 69.456; *Q* statistics (*p*) = .070; other disease: WMD = −0.378 U/L, 95% CI = −3.984 to 3.228, *p* = .837; *I* ^2^ = 92.903; *Q* statistics (*p*) ˂ .0001) participants (Table 5). Similarly, no significant impact of ACNs was discovered among the different studies in terms of intervention type as pure ACNs (WMD = −0.152 U/L, 95% CI = −2.979 to 2.675, *p* = .916) or products rich in ACNs (WMD = −1.006 U/L, 95% CI = −4.419 to 2.407, *p* = .563; Table 5). However, intake of ACNs was associated with decreased ALT levels in the studies which their primary outcomes were liver enzymes (WMD = −4.932 U/L, 95% CI = −9.848 to −0.015, *p* = .049) versus the studies which liver enzymes were their secondary outcomes (WMD = 0.297 U/L, 95% CI = −0.769 to 1.363, *p* = .387; Table 5). We observed no significant relation between dose of ACNs and ALT levels (slope = −0.004, 95% CI: −0.010 to 0.00005, *p* = .052), whereas a significant association was found between duration of trial and ALT (slope: 0.09, 95% CI = 0.040 to 0.139, *p* = .0003; Figures S2a and S3b).

**TABLE 5:** Subgroup analysis to assess the effect of supplementation with anthocyanins on liver enzymes levels

Liver enzymes	Number of trials	*I* ^2^ (%)	*Q* statistics (*p*)	WMD	95% CI	*p* effect*
ALT (U/L)						
Dose of anthocyanins						
More than 160 mg/day	7	92.872	*p* ˂ .0001	−3.358	−7.986 to 1.269	.155
160 mg/day or lower	8	13.460	0.325	0.287	−1.234 to 1.808	.712
Duration of study						
More than 56 days	8	60.651	0.013	−0.455	−2.629 to 1.719	.682
56 days or lower	7	89.933	*p* ˂ .0001	−1.279	−5.884 to 3.327	.586
Health status						
Liver disease	2	69.456	0.070	−1.728	−13.358 to 9.901	.771
Other disease	10	92.903	*p* ˂ .0001	−0.378	−3.984 to 3.228	.837
Healthy	3	62.834	0.068	−2.869	−7.701 to 1.963	.245
Type of studies in terms of assessing ALT						
Studies evaluated ALT as primary outcome	5	76.893	0.002	−4.932	−9.848 to −0.015	.049
Studies evaluated ALT as secondary outcomes	10	0.372	0.434	0.297	−0.769 to 1.363	.585
Type of intervention						
Pure anthocyanins	3	59.178	0.086	−0.152	−2.979 to 2.675	.916
Products rich in anthocyanins	12	89.022	*p* ˂ .0001	−1.006	−4.419 to 2.407	.563
AST (U/L)						
Dose of anthocyanins						
More than 160 mg/day	6	92.150	*p* ˂ .0001	−2.723	−5.880 to 0.435	.091
160 mg/day or lower	8	29.470	0.193	−0.886	−2.387 to 0.615	.247
Duration of study						
More than 56 days	7	84.771	*p* ˂ .0001	−1.769	−3.959 to 0.422	.114
56 days or lower	7	74.347	0.001	−1.578	−4.371 to 1.216	.268
Health status						
Liver disease	2	0.000	0.332	−0.608	−3.510 to 2.295	.681
Other disease	10	88.720	*p* ˂ .0001	−1.385	−3.792 to 1.021	.259
Healthy	2	74.602	0.047	−4.325	−8.516 to −0.134	.043
Type of studies in terms of assessing AST						
Studies evaluated AST as primary outcome	5	78.257	0.001	−3.464	−6.034 to −0.894	.008
Studies evaluated AST as secondary outcomes	9	43.083	0.080	−0.577	−1.957 to 0.802	.412
Type of intervention						
Pure anthocyanins	3	52.073	0.124	−0.121	−1.893 to 1.651	.894
Products rich in anthocyanins	11	79.058	*p *˂ .0001	−2.201	−4.275 to −0.127	.037
GGT (U/L)						
Dose of anthocyanins						
500 mg/day or more	2	55.526	0.134	−2.383	−7.733 to 2.967	.383
Lower than 500 mg/day	2	84.646	0.011	−4.003	−37.842 to 29.835	.817

Abbreviations: ALT, alanine aminotransferase; AST, aspartate aminotransferase; CI, confidence interval; GGT, gamma‐glutamyl transferase; WMD, weighted mean difference.

* *p* value ˂.05 considered as significant statistical level.

### 3.4 Effect of ACNs on AST

**FIGURE 3:**
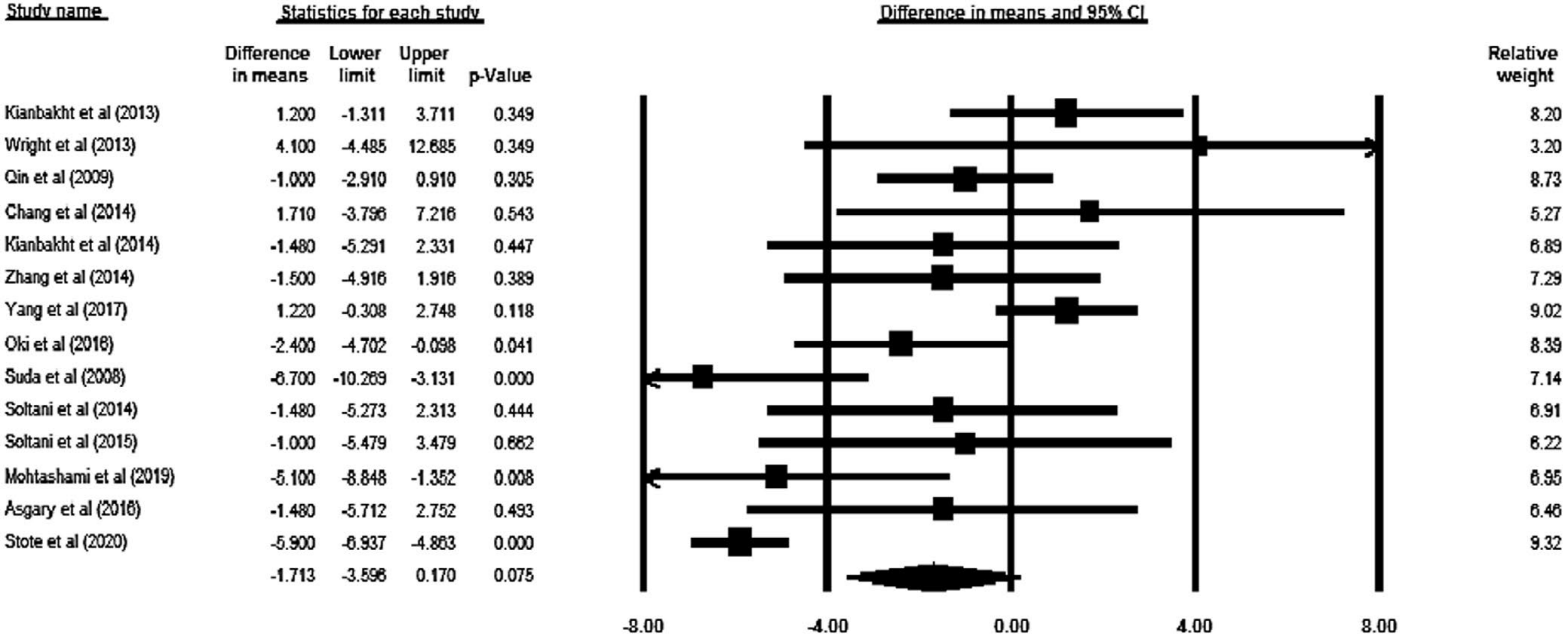
Forest plot illustrates weighted mean difference (represented by the black square) and 95% confidence interval (CI; represented by horizontal line) for Aspartate Aminotransferase (AST) concentration and anthocyanins. Weights are from random‐effects analysis. The area of the black square is proportional to the specific study weight to the overall meta‐analysis. The center of the diamond displays the pool weighted mean differences and its width shows the pooled 95% CI

Our meta‐analysis on 14 included researches (total *n* = 976, intervention: *n* = 488, placebo: *n* = 488) indicated that supplementation with ACNs had no significant impact on AST levels (WMD = −1.713 U/L, 95% CI = −3.596 to 0.170, *p* = .075; Figure 3). However, this result was close to the significant level. According to sensitivity analysis, the result of meta‐analysis was sensitive to the studies of Kianbakht et al. (2013) and Yang, Ling, Yang, et al. (2017) (Figure S4). Furthermore, a significant heterogeneity was observed across the studies (*p* < .0001, *I* ^2^ = 91.96.). Subgroup analysis showed that the impact of ACNs did not significantly differ in various doses (Dosage ˃160 mg/day: WMD = −2.723 U/L, 95% CI = −5.880 to 0.435, *p* = .091; Dosage ≤160 mg/day: WMD = −0.886 U/L, 95% CI = −2.387 to 0.615, *p* = .247; Table 5) and durations (Duration >56 days: WMD = −1.769 U/L, 95% CI = −3.959 to 0.422, *p* = .114; Duration ≤56 days: WMD = −1.578 U/L, 95% CI = −4.371 to 1.216, *p* = .268; Table 5). Consumption of ACNs was related to a decrease in AST concentrations in healthy (WMD = −4.325 U/L, 95% CI = −8.516 to −0.134, *p* = .043; *I* ^2^ = 74.602; *Q* statistics (*p*) = .047) versus unhealthy (liver disease: WMD = −0.608 U/L, 95% CI = −3.510 to 2.295, *p* = .681; *I* ^2^ = 00.00; *Q* statistics (*p*) = .332; other disease: WMD = −1.385 U/L, 95% CI = −3.792 to 1.021, *p* = .259; *I* ^2^ = 88.720; *Q* statistics (*p*) < .0001) participants (Table 5). In addition, ACNs had a significant reducing effect on AST levels in the studies which their primary outcomes were liver enzymes (WMD = −3.464 U/L, 95% CI = −6.034 to 0.894, *p* = .008) versus the studies which liver enzymes were their secondary outcomes (WMD = −0.577 U/L, 95% CI = −1.957 to 0.802, *p* = .412; Table 5). Intake of ACNs was also associated with decreased AST levels in the studies which their intervention was products rich in ACNs (WMD = −2.201 U/L, 95% CI = −4.275 to −0.127, *p* = .037) versus the studies with pure ACNs (WMD = −0.121 U/L, 95% CI = −1.893 to 1.651, *p* = .8894; Table 5). We found no significant relation between dose of ACNs and AST levels (slope = −0.002, 95% CI = −0.007 to 0.001, *p* = .197), while a significant association was observed between duration of trial and AST (slope: 0.076, 95% CI = 0.037 to 0.115, *p* = .0001; Figures S2b and S3b).

### 3.5 Effect of ACNs on GGT

**FIGURE 4:**
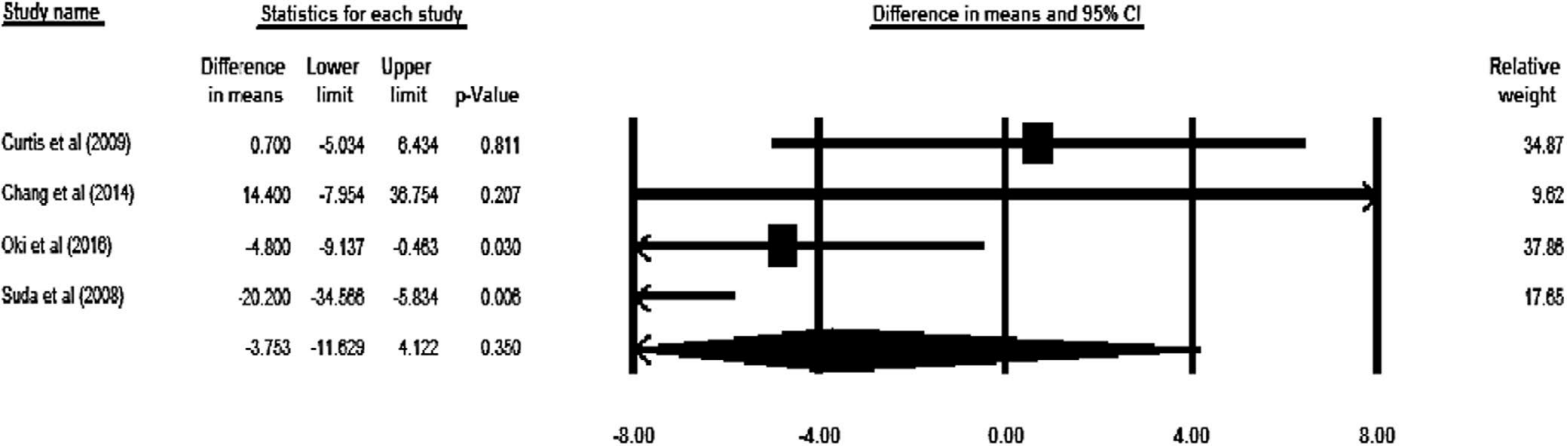
Forest plot illustrates weighed mean difference (represented by the black square) and 95% confidence interval (CI; represented by horizontal line) for Gamma‐Glutamyl Transferase (GGT) concentration and anthocyanins. Weights are from random‐effects analysis. The area of the black square is proportional to the specific study weight to the overall meta‐analysis. The center of the diamond displays the pool weighted mean differences and its width shows the pooled 95% CI

Based on the results of meta‐analysis on four included studies (*n* = 163, intervention: *n* = 83, placebo: *n* = 80), intake of ACNs had no significant impact on GGT levels (WMD = −3.753 U/L, 95% CI = −11.629 to 4.122, *p* = .350; Figure 4). In the sensitivity analysis, this result was unchanged even after removal of each study (Figure S5). A significant heterogeneity was also detected across the studies (*p* = .018, *I* ^2^ = 70.15). According to subgroup analysis, the impact of consuming ACNs was not significantly different in various doses (Dosage ˂500 mg/day: WMD = −4.003 U/L, 95% CI = −37.842 to 29.835, *p* = .817; Dosage ≥500 mg/day: WMD = −2.383 U/L, 95% CI = −7.733 to 2.967, *p* = .383; Table 5). We found no significant relation between dose of ACNs (slope = −0.020, 95% CI = −0.066 to 0.025, *p* = .384) and duration of trial (slope = 0.125, 95% CI = −0.115 to 0.366, *p* = .307) with GGT levels (Figures S2c and S3c).

### 3.6 Publication bias

According to the funnel plots and asymmetry tests, no significant publication bias was observed for the studies related to AST (Begg's test *p* = .742 and Egger'test *p* = .174) and GGT (Begg's test *p* = 1.000 and Egger test *p* = .995; Figures S6b,c respectively). Although funnel plots and Begg's test showed no significant publication bias for the studies regarding ALT (Begg's test *p* = .322), Egger test detected a publication bias (Egger test *p* = .041; Figure S6a). After adjusting the effect size for potential publication bias by the “trim and fill” correction, 42 missing studies were needed in the funnel plot that bring *p* value to ˃.05 (Adjusted values: WMD = −2.213, 95% CI = −4.894–0.468; Figure S7).

## 4 DISCUSSION

Our meta‐analysis revealed no significant effect of consuming ACNs on liver enzymes concentrations. However, intake of ACNs was significantly associated with the reduced levels of ALT and AST in the studies that evaluated liver enzymes as their primary outcomes. Moreover, ACNs had a significant decreasing effect on AST levels among healthy individuals and in the studies that used ACNs‐rich products rich as intervention. We observed no significant association between the dose of ACNs and the levels of liver enzymes. Significant associations were found between the duration of studies conducted with ALT and AST levels, while no significant relation was detected between the duration and GGT concentrations. Nevertheless, the findings of ALT should be stated carefully due to the publication bias.

In our study, sensitivity analysis indicated that the results of meta‐analysis regarding AST were sensitive to the studies conducted by Kianbakht et al. (2013) and Yang, Ling, Du, et al. (2017). In the study by Kianbakht et al. (2013), the AST levels increased markedly in the extract rich in ACNs group at the end of trial, although it was not statistically significant. Moreover, the findings of Yang, Ling, Yang, et al. (2017) showed a statistically significant increase in the AST levels in ACNs group at the end of study. These findings might be related to the results of sensitivity analysis associated with AST.

To the best of our knowledge, the current meta‐analysis is the first study on the effect of ACNs supplementation on liver enzyme. However, several systematic reviews and meta‐analysis examined the effect of ACNs on cardio‐metabolic health (Daneshzad et al., 2019; Yang, Ling, Du, et al., 2017), lipid profile (Daneshzad et al., 2019; Liu et al., 2016; Shah & Shah, 2018; Yang, Ling, Du, et al., 2017), inflammatory markers (Sangsefidi et al., 2018; Shah & Shah, 2018), and blood pressure (Zhu et al., 2016). In contrast with our findings,, other meta‐analyses reported no significant impact of ACNs on hemoglobin A1c (Daneshzad et al., 2019), C‐reactive protein (Sangsefidi et al., 2018; Shah & Shah, 2018), interleukin‐6 (IL‐6) (Shah & Shah, 2018), weight (Daneshzad et al., 2019), body mass index (Daneshzad et al., 2019), and waist circumference (Daneshzad et al., 2019). Some RCTs reported no significant impact of consuming products containing ACNs on ALT levels in healthy postmenopausal women (Curtis et al., 2009), patients with pre‐diabetes (Yang, Ling, Yang, et al., 2017), diabetes (Mohtashami et al., 2019; Soltani et al., 2015; Yang, Ling, Yang, et al., 2017), NAFLD (Chang et al., 2014), and hyperlipidemia (Kianbakht et al., 2014; Soltani et al., 2014). Similarly, intake of products containing ACNs had no significant effect on AST concentrations in patients with NAFLD patients (Chang et al., 2014; Zhang et al., 2015), pre‐diabetes (Yang, Ling, Yang, et al., 2017), diabetes (Mohtashami et al., 2019; Soltani et al., 2015; Yang, Ling, Yang, et al., 2017), and hyperlipidemia (Kianbakht et al., 2014; Soltani et al., 2014). In addition, a significant reduction was found in GGT levels following intake of the purple sweet potato extract in healthy participants with borderline hepatitis (Oki et al., 2016; Suda et al., 2008).

Similar to our findings, some meta‐analyses showed that ACNs had a significant decreasing effect on fasting blood sugar (Yang, Ling, Du, et al., 2017), homeostatic model assessment for insulin resistance (Daneshzad et al., 2019), triglyceride (Liu et al., 2016; Shah & Shah, 2018), low‐density lipoprotein (Liu et al., 2016; Shah & Shah, 2018; Yang, Ling, Du, et al., 2017), total cholesterol (Liu et al., 2016; Yang, Ling, Du, et al., 2017), tumor necrosis factor‐alpha (TNF‐α) (Shah & Shah, 2018), and blood pressure (Zhu et al., 2016). Some others reported the increasing impact of ACNs on high‐density lipoprotein (Liu et al., 2016; Shah & Shah, 2018). Moreover, intake of products containing anthocyanins resulted in a significant decrease in ALT levels in NAFLD patients (Zhang et al., 2015), healthy subjects with borderline hepatitis (Oki et al., 2016; Suda et al., 2008), and diabetic patients (Stote et al., 2020) in some RCTs. In the same vein, a significant reduction was found in AST concentrations in healthy individuals with borderline hepatitis (Oki et al., 2016; Suda et al., 2008), and diabetic patients after consumption of products containing anthocyanins. Moreover, consuming elderberry extract had no significant impact on GGT levels among healthy postmenopausal women (Curtis et al., 2009).

Discrepancies in findings of studies in this area can be attributed to various factors including discrepancies in lifestyle factors, genetic background, health status, and characteristics of participants; baseline liver enzymes levels; as well as dose and duration of consuming ACNs or sources rich in ACNs. Furthermore, discrepancies in the composition, type, and dose of ACNs in the prescribed products can cause different results. Differences in bioavailability of bioactive compounds such as ACNs due to differences in absorption, metabolism, tissue distribution, turnover, excretion, or a combination of these factors in different participants might be also resulted in various findings.

In the present meta‐analysis, among the included trials, only five studies (Chang et al., 2014; Oki et al., 2016; Stote et al., 2020; Suda et al., 2008; Zhang et al., 2015) investigated liver enzymes as their primary outcomes, while others considered liver enzymes as the secondary outcome. On the other hand, most studies were not specially designed to investigate liver enzyme. In addition, their sample size and duration may not be sufficient for assessing the factors and making concluding. As a result of these issues, some null results may be achieved. On the other hand, short duration of some trials, various doses used in interventions, and limited number of participants enrolled in the included RCTs can be mentioned to justify the null findings.

Although mechanisms regarding the effect of ACNs on liver enzymes are unclear yet, some possibilities can be presented in this regard. Evidences demonstrated that increase of the serum liver enzymes may reflect damage to the liver cells caused by some factors such as excess deposit of fat in the liver (Ahn et al., 2014), cellular oxidative stress (Suda et al., 2008), inflammation (Suda et al., 2008), and insulin resistance (Ahn et al., 2014; Bonnet et al., 2011; Zhang et al., 2010). In this regard, several animal and cell line studies showed that ACNs could reduce hepatocellular lipid accumulation via suppression of lipogenesis and promotion of lipolysis (Chang et al., 2013; Guo et al., 2011, 2012; Hwang et al., 2011; Jia et al., 2013; Peng et al., 2011; Salamone et al., 2012). ACNs also decreased hepatocellular oxidative stress by improving antioxidant responses and protection against reactive oxygen spices (Cho et al., 2011; Valenti et al., 2013; Valentová et al., 2007; Zhu et al., 2012). Furthermore, ACNs reduced cellular inflammation by suppressing NF‐κB signaling pathways (Li et al., 2017; Valenti et al., 2013), and downregulating the inflammatory genes such as TNF‐α and IL‐6 (Li et al., 2017; Valenti et al., 2013). It was also found that ACNs improved hepatic and systemic insulin resistance via up‐regulation of the glucose transporter 4 in the peripheral tissues, and activation of adenosine monophosphate‐activated protein kinase in the peripheral tissues and liver (Guo & Ling, 2015; Li et al., 2017; Valenti et al., 2013). Thus, beneficial impacts of ACNs on liver enzymes might be attributed to the protective effects of ACNs against hepatocellular lipid accumulation, oxidative stress, inflammation, and insulin resistance.

Some strengths of our study included application of a powerful search strategy without any linguistic limitations; subgroup analysis according to the health status of participants; type of studies based on liver enzymes' assessment (as primary or secondary outcomes); type of intervention (pure ACNs or products rich in ACNs); dosage of ACNs supplementation; and trial duration. Nevertheless, the current research suffered from some limitations. First, the included RCTs in our meta‐analysis had small sample sizes and limited follow‐up periods. The assessed trials were also heterogeneous in terms of various factors such as the participants' characteristics, prescription of products as pure ACNs or sources rich in ACNs, dose and duration of supplementation, as well as composition and amount of ACNs in the administered products. Moreover, the possible minor impacts of other compounds cannot be completely ignored in the sources rich in ACNs such as polyphenols, flavonoids, or other phytochemicals.

In conclusion, ACNs had no significant effect on liver enzymes levels. However, consuming ACNs was significantly related to the decrease of ALT and AST levels in the studies that assessed liver enzymes as primary outcomes. Furthermore, ACNs had a significant reducing effect on AST levels among the healthy individuals and in studies with products rich in ACNs as intervention. We found no significant relationship between the dose of ACNs and the levels of liver enzymes. Significant associations were observed between the duration of studies with ALT and AST levels, while no significant relation was discovered between duration of trials and GGT concentrations. However, the findings related to ALT should be reported and applied carefully due to the publication bias. Further well‐designed RCTs are required with larger sample sizes and longer follow‐ups to confirm these findings.

## CONFLICT OF INTERESTS

The authors declare that there are no competing interests.

## AUTHOR CONTRIBUTIONS

**Zohreh Sadat Sangsefidi:** Data curation (equal); Formal analysis (supporting); Investigation (equal); Methodology (supporting); Project administration (equal); Writing‐original draft (equal). **Hassan** **Mozaffari‐Khosravi:** Conceptualization (lead); Investigation (supporting); Methodology (supporting); Writing‐review & editing (supporting). **Sahar** **Sarkhosh‐Khorasani:** Data curation (equal); Investigation (equal); Project administration (equal); Writing‐original draft (equal). **Mahdieh Hosseinzadeh:** Conceptualization (lead); Formal analysis (lead); Investigation (lead); Methodology (lead); Writing‐review & editing (lead).

## Supporting information

Fig S1

Fig S2

Fig S3

Fig S4

Fig S5

Fig S6

Fig S7

## Data Availability

All data generated or analyzed during this study have been included in this published article.
