# Supplementary material for: The effect of anthocyanins supplementation on liver enzymes: A systematic review and meta‐analysis of randomized clinical trials
Source: Food Sci Nutr. 2021 May 6;9(7):3954–70. doi: 10.1002/fsn3.2278 (PMC8269574; doi:10.1002/fsn3.2278)
Supplement: Supplementary file 2 — Fig S2 [file FSN3-9-3954-s005.docx]

**A**

**B**

**C**

**Supplementary figure 2.** Meta-regression plots of the association between weighted mean difference in liver enzymes levels values (A: Alanine aminotransferase, B: Aspartate aminotransferase, C: Gamma-glutamyl transferase) after anthocyanins consumption with dose of anthocyanins. The size of each circle is inversely proportional to the variance of change.
