# Supplementary material for: The effect of anthocyanins supplementation on liver enzymes: A systematic review and meta‐analysis of randomized clinical trials
Source: Food Sci Nutr. 2021 May 6;9(7):3954–70. doi: 10.1002/fsn3.2278 (PMC8269574; doi:10.1002/fsn3.2278)
Supplement: Supplementary file 5 — Fig S5 [file FSN3-9-3954-s004.docx]

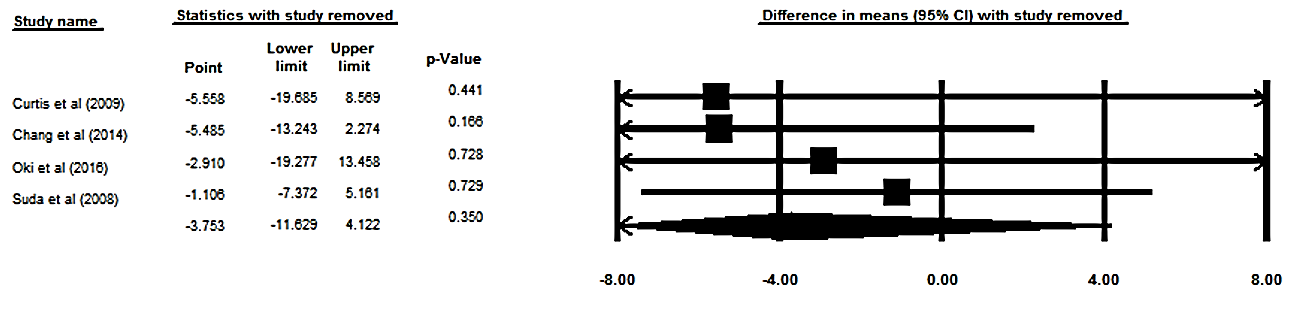


**Supplementary figure 5.** Sensitivity analysis was performed using a random-effect model for impact of anthocyanins and Gamma-Glutamyl Transferase (GGT) concentration.
