# Supplementary material for: The effect of anthocyanins supplementation on liver enzymes: A systematic review and meta‐analysis of randomized clinical trials
Source: Food Sci Nutr. 2021 May 6;9(7):3954–70. doi: 10.1002/fsn3.2278 (PMC8269574; doi:10.1002/fsn3.2278)
Supplement: Supplementary file 6 — Fig S6 [file FSN3-9-3954-s003.docx]

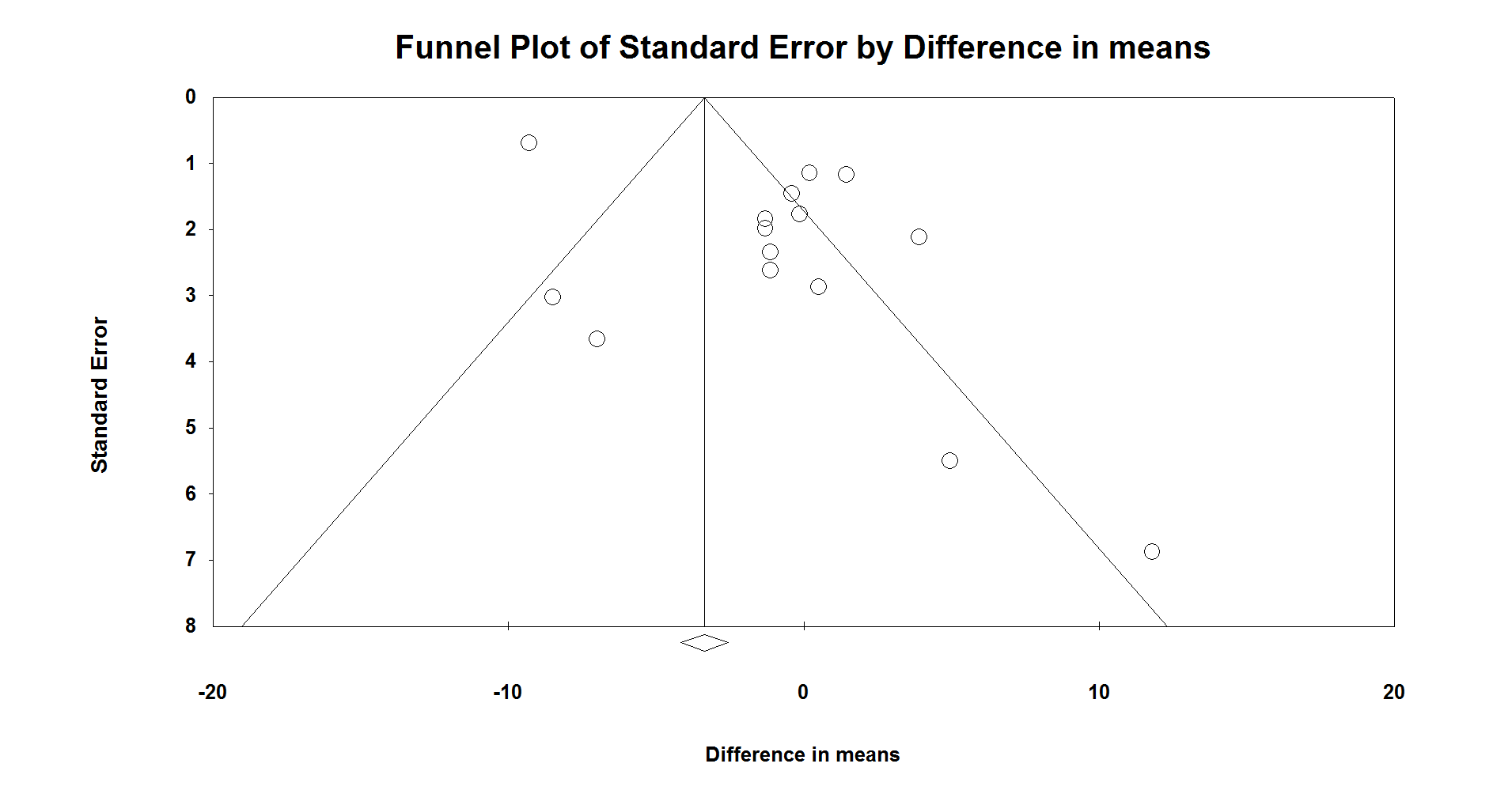


**A**


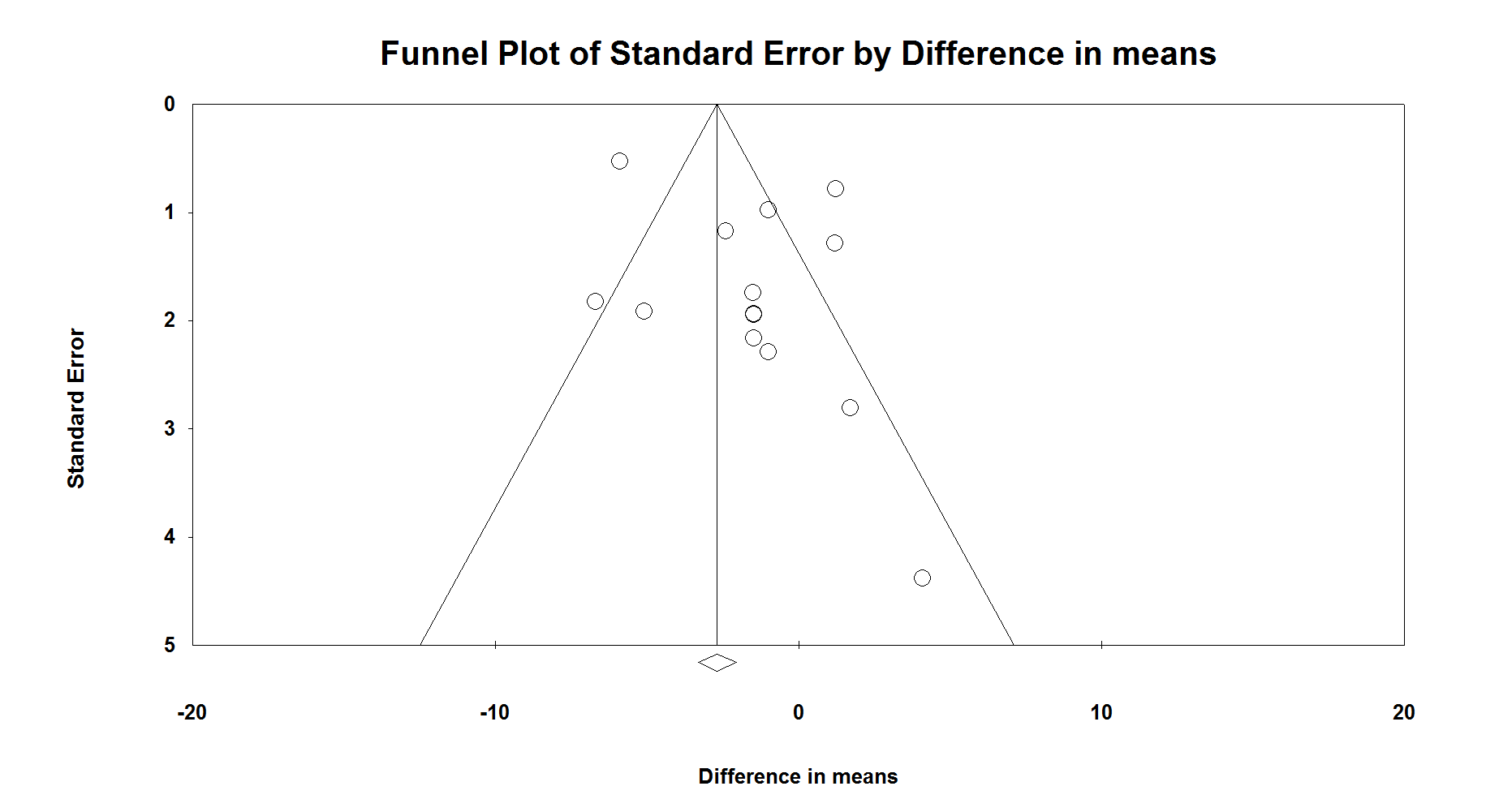


**B**


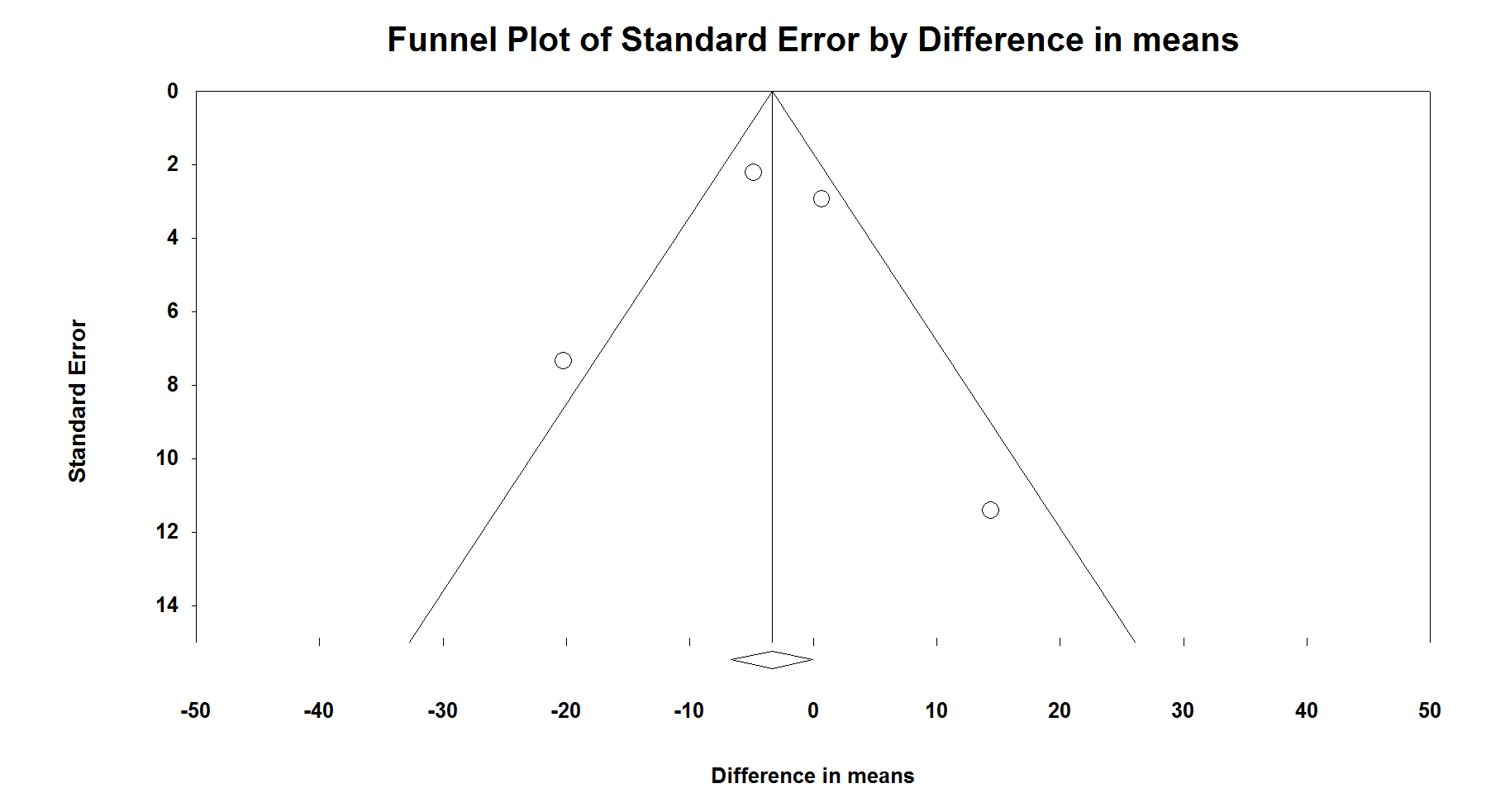


**C**

**Supplementary figure 6**. Funnel plot (with 95 % confidence intervals) of weighted mean differences for liver enzymes (A: Alanine Aminotransferase (ALT), B: Aspartate Aminotransferase (AST), C: Gamma-Glutamyl Transferase) concentrations with anthocyanins. The vertical line (——) shows the combined mean differences calculated with the random-effects model.
