# Supplementary material for: The effect of anthocyanins supplementation on liver enzymes: A systematic review and meta‐analysis of randomized clinical trials
Source: Food Sci Nutr. 2021 May 6;9(7):3954–70. doi: 10.1002/fsn3.2278 (PMC8269574; doi:10.1002/fsn3.2278)
Supplement: Supplementary file 7 — Fig S7 [file FSN3-9-3954-s001.docx]

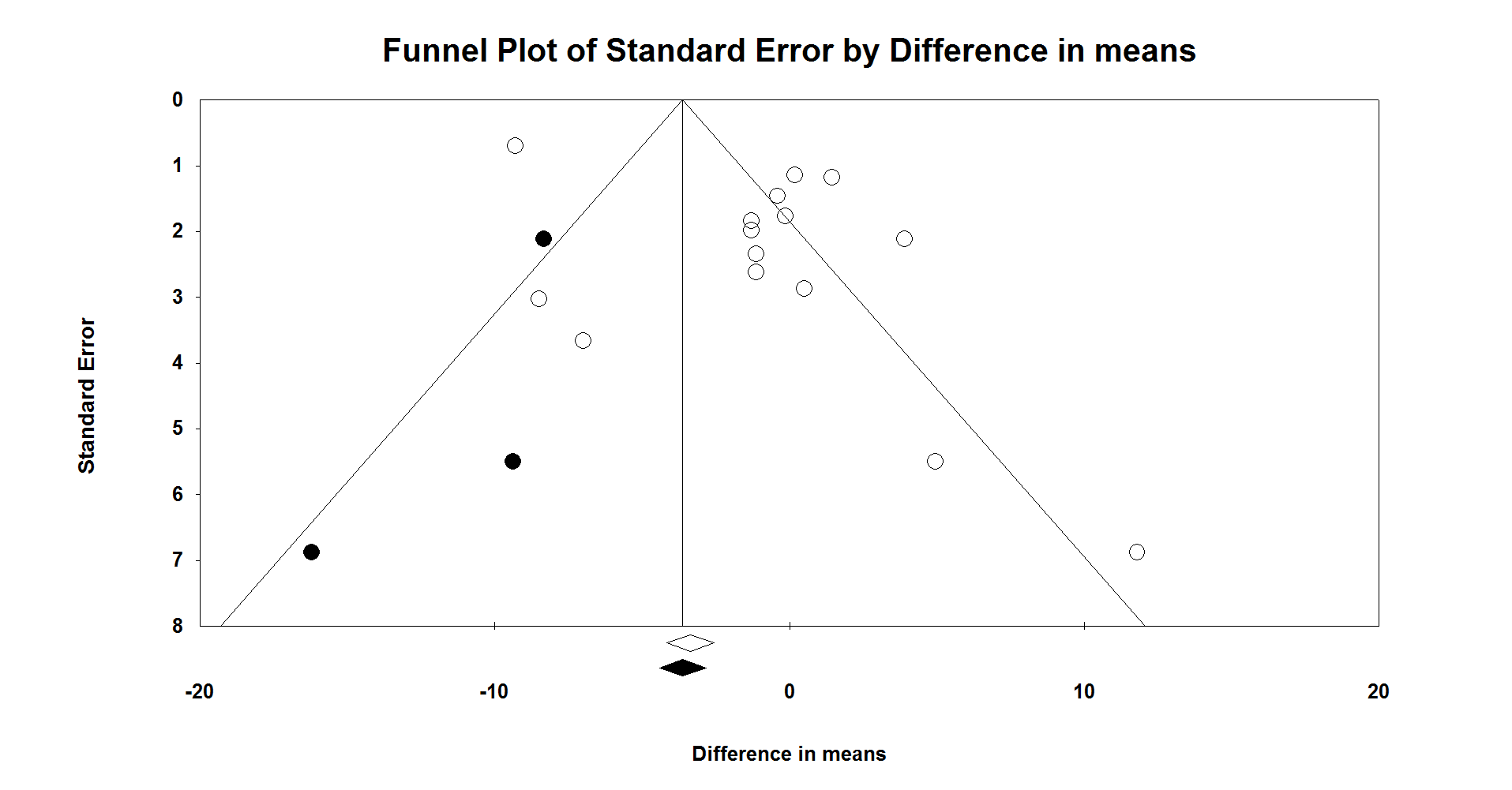


**Supplementary figure 7.** Trim and fill method was used to impute for potentially missing studies for alanine aminotransferase (ALT), open circles represent observed published studies; open diamond represents observed effect size; closed diamond represents imputed effect size; closed circles represents imputed studies.
